# Investigation of stent retriever removal forces in an experimental model of acute ischemic stroke

**DOI:** 10.3389/fneur.2024.1486738

**Published:** 2024-10-31

**Authors:** Demitria A. Poulos, Michael T. Froehler, Bryan C. Good

**Affiliations:** ^1^Department of Mechanical, Aerospace, and Biomedical Engineering, University of Tennessee, Knoxville, TN, United States; ^2^Cerebrovascular Program, Vanderbilt University Medical Center, Nashville, TN, United States

**Keywords:** mechanical thrombectomy, stent retriever, tortuosity, *in vitro* model, acute ischemic stroke

## Abstract

**Introduction:**

Mechanical thrombectomy becomes more complex when the occlusion occurs in a tortuous cerebral anatomy, increasing the puncture to reperfusion time and the number of attempts for clot removal. Therefore, an understanding of stent retriever performance in these locations is necessary to increase the efficiency and safety of the procedure. An *in vitro* investigation into the effects of occlusion site tortuosity, blood clot hematocrit, and device geometry was conducted to identify their individual influence on stent retriever removal forces.

**Methods:**

Embolus analogs were used to create occlusions in a mock circulatory flow loop, and *in vitro* mechanical thrombectomies were performed in arterial models of increasing tortuosity. The stent retriever removal forces of Solitaire Platinum and EmboTrap II devices were recorded through each geometry with and without embolus analogs present. Similar experiments were also conducted with Solitaire stent retrievers of varying lengths and diameters and 0, 25, and 50% hematocrit embolus analogs.

**Results:**

The removal force increased as model tortuosity increased for both the Solitaire Platinum and EmboTrap II stent retriever devices. The average removal forces in the simplest geometry with the Solitaire Platinum and EmboTrap II were 0.24 ± 0.01 N and 0.37 ± 0.02 N, respectively, and increased to 1.2 ± 0.08 N and 1.6 ± 0.17 N, respectively, in the most complex geometry. Slight increases in removal force were found with 0% hematocrit embolus analogs, however, no statistical significance between removal force and EA hematocrit was observed. A comparison between stent retriever removal forces between devices of different diameters also proved to be significant (*p* < 0.01), while forces between devices of varying lengths were not (*p* > 0.05).

**Conclusion:**

Benchtop mechanical thrombectomies performed with commercial stent retrievers of varying geometry showed that device removal forces increase with increasing model tortuosity, clot hematocrit does not play a significant role in device removal force, and that a stent retriever’s diameter has a greater impact on removal forces compared to its length. These results provide an improved understanding of the overall forces involved in mechanical thrombectomy and can be used to develop safer and more effective stent retrievers for the most difficult cases.

## 1 Introduction

Stroke is one of the leading causes of long-term disability worldwide (1). In the United States, 87% of stroke cases are classified as ischemic (2), resulting from the lack of blood flow to the brain. Acute ischemic stroke (AIS) due to large vessel occlusions (LVOs), such as occlusions of the internal carotid artery and M1 and M2 segments of the middle cerebral artery, account for 90% of 6-month post stroke mortality (3). Fast recanalization, restoration of cerebral blood flow, is key to maintaining neurologic function and decreasing the chance of long-term complications and morbidity in stroke patients (4, 5). Tissue-plasminogen activator (tPA) has been a standard form of stroke treatment since 1995 but is most beneficial when administered within 4.5 h of stroke onset (3). Mechanical thrombectomy (MT), a transfemoral or transradial endovascular approach (6, 7), can increase the treatment window from 4.5 to 6–24 h (8), and has proven to be a safe and more effective recanalization treatment for AIS when compared to drug therapy alone (9). To achieve recanalization, current MT procedures require the use of aspiration or stent retriever (SR) techniques.

Aspiration thrombectomy utilizes a large-bore catheter to apply suction to the proximal face of the thrombus, while SR thrombectomy involves the deployment of a stent across the entire thrombus and the system is retracted to achieve recanalization (10). A meta-analysis comparing the efficacy outcomes of SR and direct aspiration identified no significant difference in good clinical outcomes and mortality at 3 months (11), and a study comparing SR and contact aspiration concluded there was no difference in recanalization rates between these first line approaches (12). The choice of treatment is ultimately based on device availability and surgeon preference (12); however, a study has shown that rescue therapies are more often needed with direct aspiration thrombectomies than with SR thrombectomies (12). Despite the clinical success of SR MT, adverse outcomes including hemorrhage and thrombus embolization to distal vessels can occur (13). This procedure becomes more complex when the occlusion occurs in a difficult cerebral anatomy, including distal arteries and those with severe tortuosity (14). An analysis of 592 AIS patients was able to link a third of thrombectomy failures to vessel tortuosity (15). These difficult AIS anatomies can delay recanalization by increasing the puncture to reperfusion time, as well as increasing the number of attempts for blood clot removal (16). Patients with tortuous cerebral anatomies are also at a higher risk of hemorrhaging after MT (16).

Understanding the performance of SRs in tortuous anatomies is crucial to decreasing procedure times and complication rates. A component of this is understanding the mechanical forces involved in blood clot dislodgment during SR MT. Benchtop circulatory flow systems have been used to investigate the deformability of SRs (17, 18), as well as the effect of embolus analog (EA) hematocrit on their fracture and embolization (19, 20) during MT. AIS experimental studies rarely include an evaluation of the SR removal force (i.e., the maximum force required to pull the MT device through the occlusion site), and limited information is available regarding SR MT in tortuous cerebral geometries. Studies with multiple SRs reported that longer and larger diameter SRs had the highest degree of first pass effect [i.e., removal of the thrombus with one pass (FPE)] (21–23). It is suggested that these larger diameter SRs produce a greater radial force and therefore promote integration between the SR and thrombus (21, 22), but this can also cause vasospasms and vessel irritation (21, 24). Therefore, we seek to improve the safety and degree of revascularization for SR MT by investigating the individual effects of occlusion site tortuosity, EA hematocrit, and SR geometry on the SR removal force.

## 2 Materials and methods

### 2.1 Embolus analog fabrication

Whole bovine blood was obtained from a third-party supplier (Lampire Biologic) in citrate phosphate dextrose adenine (CPDA-1) anticoagulated bags. The blood was centrifuged to separate red blood cells (RBCs) and platelet rich plasma (PRP), reconstituted to achieve a specific blood hematocrit (0, 25, or 50%), and then recalcified with calcium chloride (CaCl_2_) to promote coagulation at a concentration of 20 mM in blood. The reconstituted and recalcified blood was injected into 5.5 mm diameter tubing and placed in a dynamic Chandler Loop system, where they rotated at 30 RPM to mimic cerebral flow conditions [approximately 240 mL/min through the ICA (25)]. EAs were allowed to form under dynamic flow conditions at body temperature (37°C) for approximately 2 h. After the clotting period, the EAs were removed from the Chandler Loop and stored at 4°C in PBS for up to 2 days until experimental MT testing. The EAs were fabricated from multiple bovine blood donors for each experiment to account for variability in their blood compositions.

### 2.2 *In vitro* AIS and MT modeling

**Figure 1:**
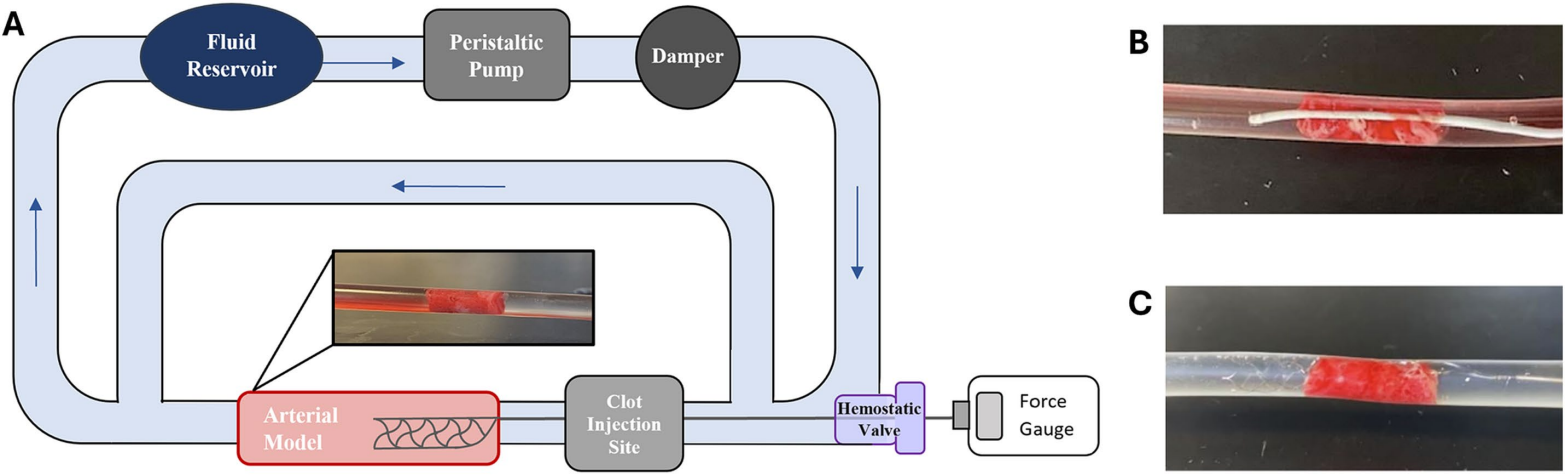
**(A)** Schematic of AIS circulatory flow loop used for SR MT testing. **(B)** Example of a Phenom21 (Medtronic) microcatheter passed across a lodged EA and subsequent. **(C)** Solitaire Platinum (Medtronic) SR deployment.

To experimentally study AIS, a benchtop circulatory flow loop (Figure 1A) was developed with a peristaltic pump, fluid reservoir filled with water, and a cerebral artery model. To mimic AIS and MT, an EA was injected upstream in the circulatory flow loop and was allowed to lodge downstream within the arterial model for 5 min (Figure 1A). After this period, which ensured EA lodging, a SR within a microcatheter was inserted through a hemostatic valve and completely passed across the EA from the upstream to the downstream direction (Figure 1B), and the SR was deployed (Figure 1C). The SR was then connected to a force gage (MXmoonfree) attached to a syringe pump (kdScientific), which functioned as a stepper motor to pull the SR with the EA. This ensured the SR was pulled at a constant speed of 2 mm/s [like Machi et al. (18)] for 15 s while simultaneously recording the maximum SR removal force during the testing period. Control tests, SR flow loop experiments performed without EAs, were also conducted to collect baseline force measurements for experimental variables. For the control tests, a SR was deployed within an arterial model and attached to the force gage, as in previously stated protocols, and the maximum SR removal force was recorded over a 15 s pull period.

**Figure 2:**
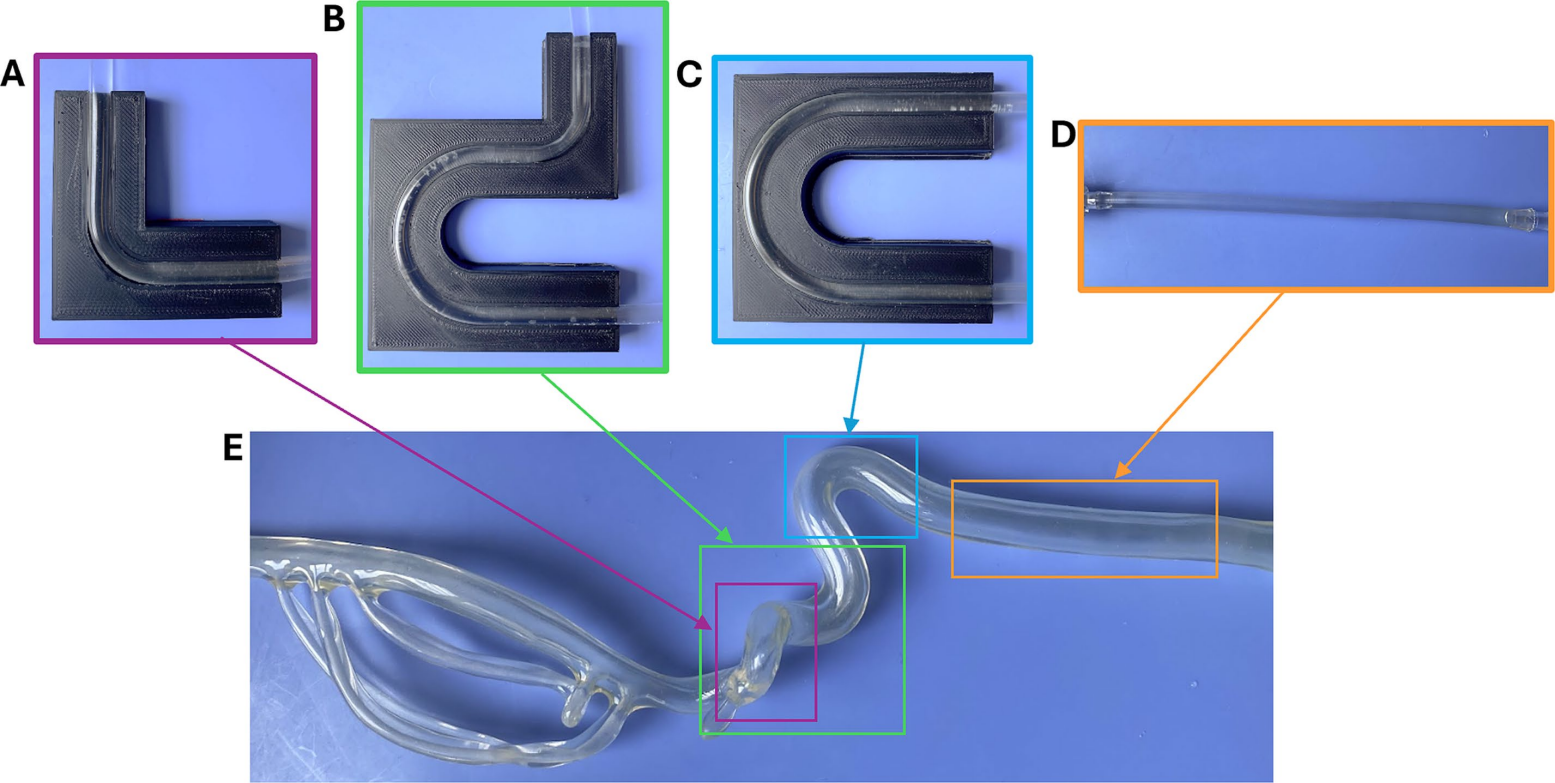
**(A)** Right angle, **(B)** 3-bend, **(C)** U-shape, and **(D)** straight geometries used in the flow loop, and segments of the **(E)** silicone ICA/MCA model (United Biologics) that inspired them.

The arterial model of the flow loop was interchanged with four simplified geometries inspired by segments of a United Biologics silicone ICA/MCA model (Figure 2E). The silicone model was separated into straight (Figure 2D), right-angle (Figure 2A), U-bend (Figure 2C), and 3-bend (combination of the right-angle and U-bend geometries) (Figure 2B) geometries, to model common sites of AIS occlusion. To represent tapering that occurs in cerebral arteries, and to ensure each EA lodged within the flow loop, the arterial models were tapered along their lengths from 5.5 to 3 mm in diameter. These simple vascular models were constructed from PVC tubing, and PLA molds were printed with an Ultimaker S3 3D-printer to maintain their shapes during MT testing.

### 2.3 Investigation into SR-MT parameters

**Figure 3:**
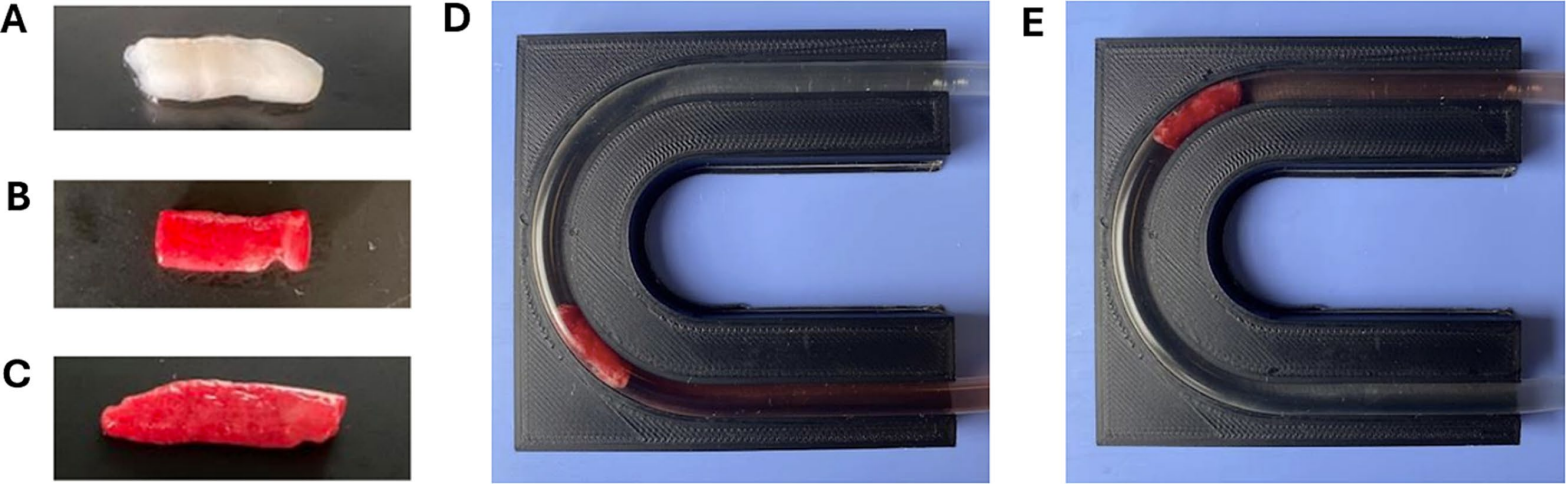
Dynamically formed **(A)** 0%, **(B)** 25%, and **(C)** 50% hematocrit EAs used in SR MT flow loop testing. Examples of 50% hematocrit EAs lodged in **(D)** U-1 and **(E)** U-2 locations of the U-shape model, respectively.

To observe the effect of occlusion site geometry on the SR removal force, all cerebral artery models (straight, right-angle, U-shape, and 3-bend) were individually evaluated while holding all other variables constant. The U-shape model was also separated into proximal (U-1) (Figure 3D) and distal (U-2) (Figure 3E) bend occlusion locations for testing, therefore SR removal force data was collected from five different arterial models. Each of the geometries were tested within the arterial model segment of the flow loop shown in Figure 1A, and the previously stated flow loop and SR MT protocols were followed to measure the SR removal force of clots lodged in each location. Control tests were also performed in each geometry with 4 × 20 mm Solitaire Platinum (Medtronic, Minneapolis, MN, United States) and 5 × 21 mm EmboTrap II (Cerenovus, Irvine, California, United States) SR devices. The SR removal force tests through each geometry with EAs present were only conducted using the Solitaire Platinum SR and 50% hematocrit EAs. A sample size of *n* = 20 was consistent across all geometries.

**Figure 4:**
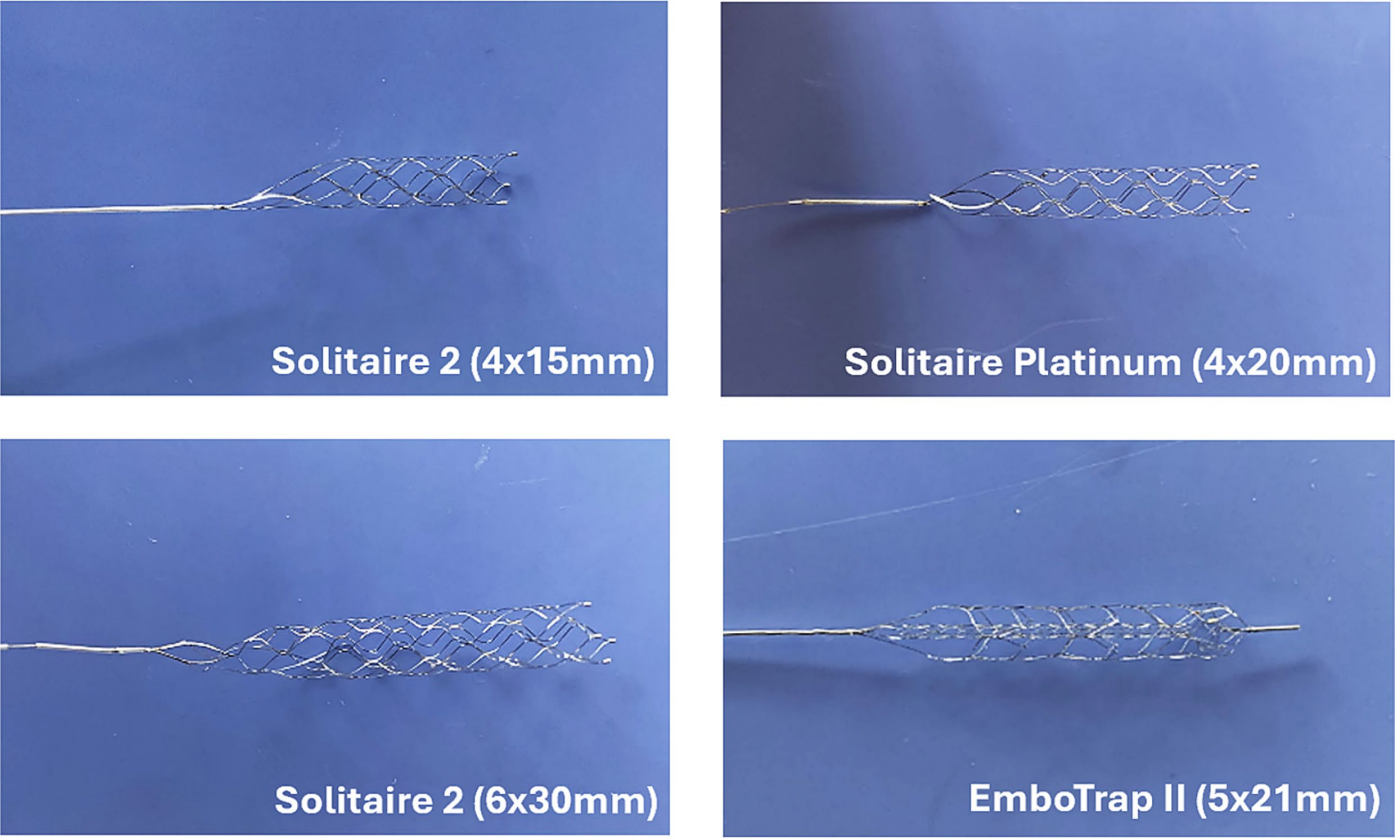
Images of Solitaire (Medtronic) and EmboTrap (Cerenovus) stent retrievers used in experimental flow loop testing.

Additional flow loop tests were performed using the straight tube arterial model and various SRs and EA hematocrits. The motivation for these experiments was to determine the influence of EA hematocrit and SR geometry on the SR removal force. EAs of 0, 25, and 50% hematocrit (Figures 3A-C) were fabricated for testing with various SRs. The SRs compared in these experiments were the 4 × 20 mm Solitaire Platinum, 4 × 15 mm Solitaire 2, and 6 × 30 mm Solitaire 2 (Figure 4). The Soliatire2 and Solitaire Platinum are nearly identical in their overall designs [the Solitaire 2 is the predicate of the Solitaire Platinum design (26)], with the major difference being the placement of radiopaque markers on the Solitaire Platinum and its approval for use in various vessels (26). Therefore, in the context of our experiments, we believe that force results can be compared across all three of the tested SRs. Similar to the previous set of experiments, a sample size of *n* = 20 was used for each hematocrit (*n* = 60 for each SR).

### 2.4 Statistical analysis

A one-way ANOVA (*p* < 0.05) with a Tukey *post hoc* test (*α* = 0.05) was used to assess the differences in SR removal force between various arterial models, EA hematocrits, and SR geometries (260 SR MT experiments in total). The length and diameter of each EA was measured using digital calipers prior to testing and ranged between 12.8–18 mm in length, and 4.8–6.5 mm in diameter. These EA dimensional variables were also evaluated relative to their respective SR removal forces but with no significant differences found. Thus, EA length and diameter were not considered as factors in the following analysis.

## 3 Results

**Figure 5:**
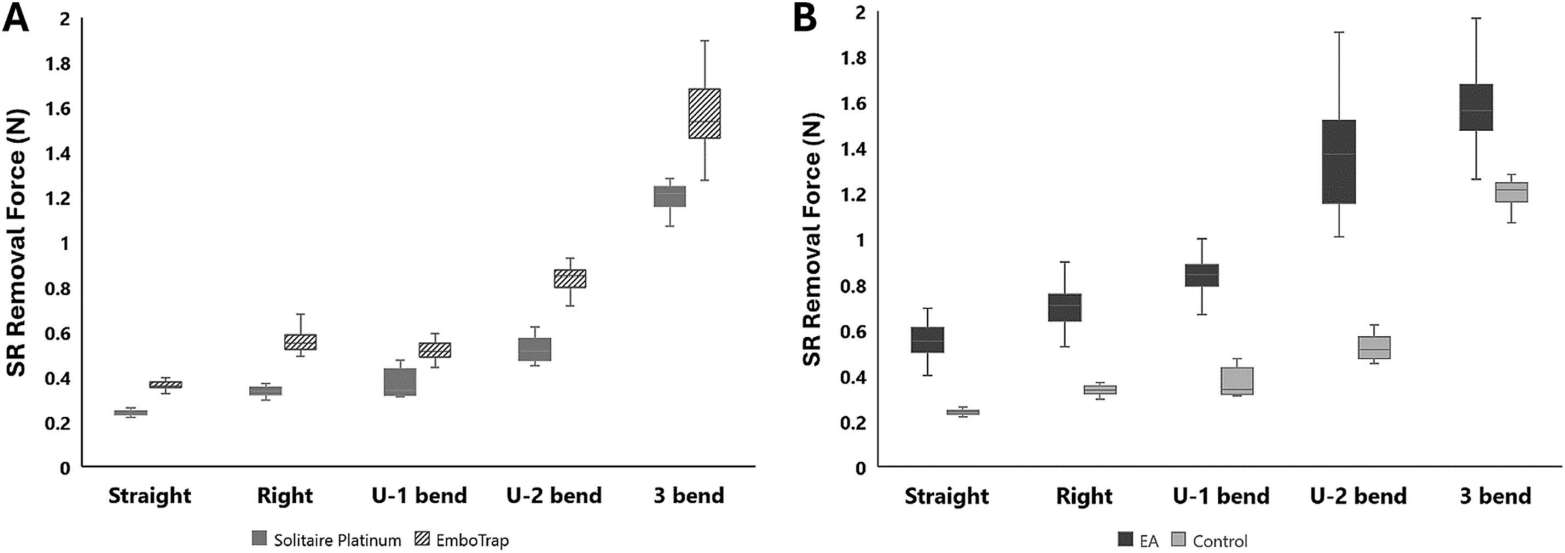
**(A)** Control test data with the Solitaire Platinum and EmboTrap II SR devices. **(B)** A comparison of control and 50% hematocrit EA removal force data with the Solitaire Platinum SR.

Control tests were performed in the straight, right-angle, U-bend, and 3-bend arterial models with both the Solitaire Platinum and EmboTrap SRs, while removal tests were performed with 50% hematocrit EAs in each geometry with the Solitaire Platinum SR (Table 1). As seen in Figure 5, the SR removal force increased with model tortuosity whether occluded EAs were present or not. For the control data, the average SR removal forces in the simplest geometry (straight tube) were 0.24 ± 0.01 N and 0.37 ± 0.02 N for the Solitaire Platinum and EmboTrap II devices, respectively. With the most complex geometry (3-bend), the average SR removal forces were 1.2 ± 0.08 N and 1.6 ± 0.17 N for the Solitaire Platinum and EmboTrap II devices, respectively. Analysis of the control data for the EmboTrap II indicated statistical differences in SR removal force between all arterial models (*p* < 0.01), except between the right angle and U-1 geometries. The same was observed with the Solitaire Platinum control data, with statistical differences found in SR removal forces among all models (*p* < 0.01), except between the right angle and U-1 geometries. Solitaire Platinum removal force data collected with lodged 50% hematocrit EAs is displayed in Figure 5B. When compared to the Solitaire Platinum control data (Figure 5A), the SR removal forces nearly doubled across all geometries with the presence of an EA. The only exception to this was the force measured in the 3-bend model, which increased by approximately 36% when an EA was present.

**Table 1:** Average SR removal forces of the EmboTrap II and Solitaire Platinum in different arterial geometries.

		SR removal force (N)				
SR	EA hematocrit (%)	Straight	Right	U-1	U-2	3-bend
EmboTrap II (5 × 21)	**–**	0.37 ± 0.02	0.56 ± 0.05	0.53 ± 0.06	0.84 ± 0.06	1.55 ± 0.17
Solitaire platinum (4 × 20)	**–**	0.24 ± 0.01	0.35 ± 0.06	0.37 ± 0.06	0.52 ± 0.05	1.20 ± 0.08
Solitaire platinum (4 × 20)	50	0.56 ± 0.07	0.7 ± 0.09	0.86 ± 0.11	1.34 ± 0.24	1.63 ± 0.36

**Figure 6:**
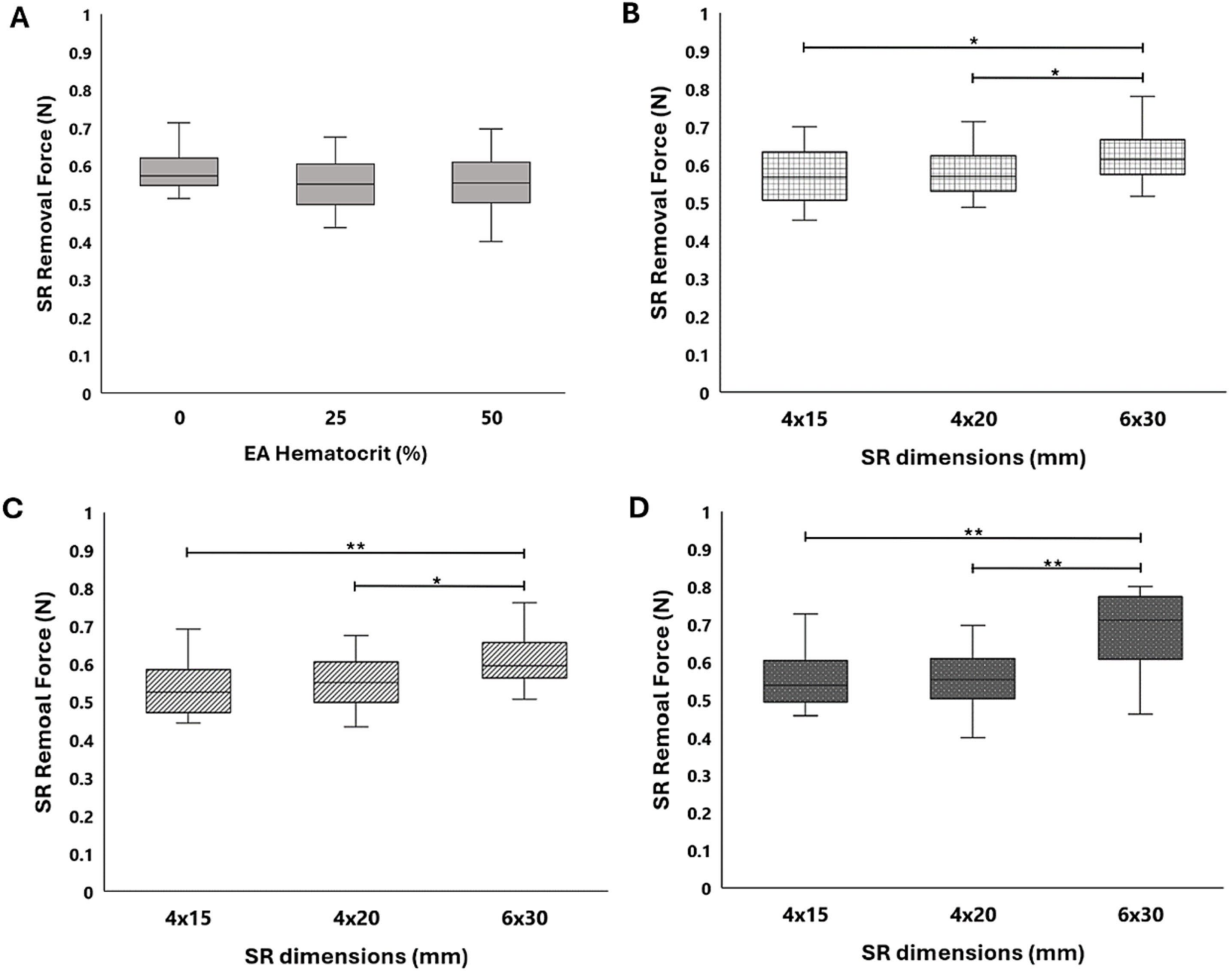
**(A)** SR removal force data collected in a straight tube with 0, 25, and 50% hematocrit EAs for the Solitaire Platinum SR. Comparison of force data across the Solitaire Platinum (4 × 20 mm), Solitaire 2 (4 × 15mm), and Solitaire 2 (6 × 30 mm) SRs for **(B)** 0%, **(C)** 25%, and **(D)** 50% hematocrit EAs. A significance of *p* < 0.05 is denoted by (*) and significance of *p* < 0.01 is denoted by (**).

The results from all flow loop experiments in the straight tube (average SR removal force) with various EA hematocrits and SRs are displayed in Table 2. A slight increase in force was present with the 0% hematocrit EAs, as seen in Figure 6A, yet a statistical significance between removal force and EA hematocrit was not observed. This was expected based on the lower clinical removal rate of fibrin rich clots (27). A similar trend was observed with the Solitaire 2 4 × 15 mm and 6 × 30 mm devices. Flow loop SR MT experiments were first performed with SRs of similar diameters and varying lengths within the straight tube model. A comparison of force data through a one-way ANOVA with Tukey’s *post hoc* test for the Solitaire Platinum (4 × 20 mm) and Solitaire 2 (4 × 15 mm) for each hematocrit proved insignificant. These results were then compared to the SR removal force data collected with the 6 × 30 mm Solitaire 2. An analysis for each EA hematocrit indicated a statistical significance (*p* < 0.01) in force between SRs of varying diameters (Figures 6B–D).

**Table 2:** Average SR removal force with various SRs and EA hematocrits in the straight tube geometry.

SR	EA hematocrit (%)	SR removal force (N)
Solitaire platinum (4 × 20)	0	0.57 ± 0.08
	25	0.55 ± 0.07
	50	0.56 ± 0.07
Solitaire 2 (4 × 15)	0	0.57 ± 0.07
	25	0.54 ± 0.07
	50	0.55 ± 0.07
Solitaire 2 (6 × 30)	0	0.63 ± 0.07
	25	0.62 ± 0.09
	50	0.69 ± 0.09

## 4 Discussion

**Figure 7:**
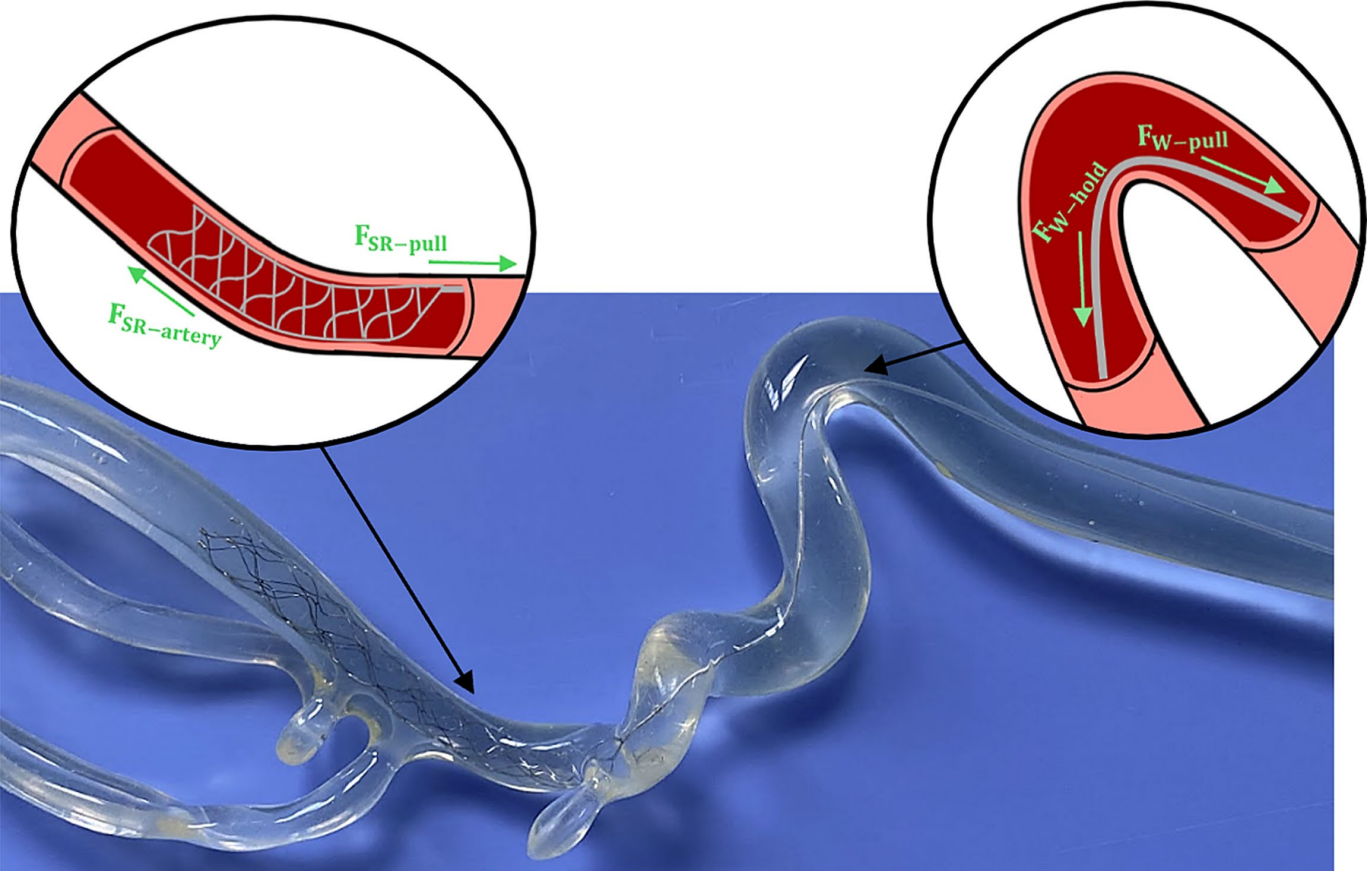
Example of SR deployment within an ICA/MCA model with schematics highlighting the individual forces experienced by the SR body and pushwire during retrieval.

For this study, the SR removal force was identified as the maximum pulling force required to fully move the SR from the occlusion site. Here we assume that the SR removal force is a combination of the forces experienced by SR body and pushwire, as shown in Figure 7. To remove a lodged thrombus and restore blood flow, the deployed SR must overcome additional resistive forces (i.e., friction and adhesion between the thrombus and vessel wall, and blood pressure on the thrombus’ proximal face). Therefore, the applied pulling force of the SR by the clinician (28) will be influenced by both the tortuosity of the local arterial occlusion site (28, 29) and the friction between the pushwire and vessel wall. The pushwire frictional forces can described by the capstan equation (30, 31), which relates the tensile force across a rope over a curved surface (Equation 1):

(1) $$F_{W,\;\mathrm{hold}}=F_{W,\mathrm{pull}}e^{\mu \varphi }$$

where $F_{W,\mathrm{hold}}$ is the holding force resisting pushwire motion, $F_{W,\mathrm{pull}}$ is the pulling force on the pushwire during retrieval, μ is the coefficient of friction between the pushwire and vessel wall, and φ is the pushwire wrap angle (i.e., angle of contact between the pushwire and vessel wall).

Clinical and experimental studies have shown that SRs can underperform or cause complications in tortuous anatomies (32, 33), leading to higher rates of failed MT. Constant SR to vessel wall interaction has been identified as a key factor for successful recanalization (32, 34), but when current SRs are pulled in extremely tortuous locations they can elongate and lose contact with the arterial wall (18). Multiple SR attempts are then required to remove the thrombus, which can damage the vessel and increase the procedure time (35). Ideally the thrombus is removed during the first SR pass (i.e., FPE), restoring blood flow to the patient as soon as possible (36), but this is not always guaranteed. Investigating the SR removal force brings us closer to understanding the overall forces involved in MT and potentially increasing rates of FPE. An improved understanding of the parameters necessary for blood clot dislodgement can also lead to the development of safer and more effective SRs.

Previous experimental studies of AIS and MT have included realistic arterial models that require both the EA and SR to pass through multiple complex anatomical features during retrieval (19, 28). Kwak et al. (28) theorized that the curvature of the arteries influenced the SR pulling force, but this was not further investigated in their work. In the present study, a realistic ICA/MCA anatomy was segmented into four simplified anatomical regions (Figure 2) such that the effects of AIS location and local arterial geometry on SR removal force could be identified in isolation. Five occlusion locations were used in total (both proximal, U-1, and distal, U-2, locations within the U-shape model) with increasing levels of tortuosity. Through this investigation, it was determined that SR removal force increased with model complexity and tortuosity, with the lowest removal forces found in the straight tube model and the highest in the 3-bend model for all tested SRs (Figure 5). Investigating the SRs pathway through the cerebral arteries as it is deployed and retracted is still a new topic (37). Based on prior work that linked tortuosity to lower rates of MT success (32), we hypothesize that a greater removal force is needed to achieve revascularization in tortuous segments. To better quantify this relationship, the tortuosity index (TI), a ratio used clinically to measure the tortuosity of vascular segments [Equation 2; (38)], was calculated for each of the five models:

(2) $$TI=\frac{L_{1}}{L_{2}}$$

where $L_{1}$ is the length along the centerline of the vessel and $L_{2}$ is the linear distance between the start and end points of the vessel. The TI of the right angle geometry was 1.15, while the TI of the U-1 geometry was 1.25. This is in comparison to TIs of 1.61 for the U-2 geometry and 2.12 for the 3-bend geometry. We suspect the removal force data between the right angle and U-1 geometries were not significantly different due to their similar TI values.

Clinical studies have also shown that clot hematocrit can influence the outcomes of MT (39), while experimental studies have determined that lower hematocrit (fibrin rich) EAs have higher frictional coefficients and are therefore more likely to resist removal (40). However, limited research is available on EA hematocrit and EA adhesion within *in vitro* AIS models, which typically include arterial models made of PVC, glass, or silicone (17, 19, 22). Therefore, we chose to investigate the influence of EA hematocrit on SR removal forces in a simplified straight tube model. Through this evaluation, it was determined that EA hematocrit does not significantly influence SR removal force. It should be taken into consideration, however, that the arterial models for this setup were composed of PVC tubing. While this material mimics the flexibility of arteries, its surface properties cannot be compared to the endothelium of cerebral arteries. Differences have been observed in frictional properties between the vessel material walls and nitinol SRs and in the adhesive behaviors of the EA in the model (40). This study utilized a Chandler loop system to fabricate EAs under dynamic flow conditions similar to those in the carotid artery (41). Through this method, dynamically formed EAs were composed of a fibrin matrix with patches of RBCs distributed throughout. This heterogeneous composition produces firmer EAs that closely mimic those removed through MT from AIS patients (41, 42).

Control tests were performed using both the Solitaire Platinum and EmboTrap II to determine removal force differences related to SR device design (Table 1; Figure 5A). The Solitaire Platinum is considered to be part of the “2nd Generation” of MT devices, which first introduced SRs, while the EmboTrap II is part of the new “3rd Generation” of devices (43, 44). The first class of SRs combined stent scaffolds with retrieval techniques (43) and consisted of a single-layer design with a closed cell structure and open tip (44, 45), as seen with the Solitaire devices in Figures 4A–C. The newest generation of SRs, which includes the EmboTrap, is focused on increasing the FPE, further reducing distal embolization, and improving safety. These devices consist of segmented or compartmental designs, a combination of open and closed cell structures and a closed tip (44, 45). The EmboTrap II, specifically, has a dual-layer design and mesh closed tip (46) (Figure 4D). In comparing the control test between the Solitaire Platinum and EmboTrap II (Table 1; Figure 5A), the EmboTrap II was found to require higher removal forces for all tested models. This was not surprising due to the fact that the EmboTrap II has a larger diameter than the Solitaire Platinum (5 mm versus 4 mm), and the significant effect SR diameter had on removal forces found between Solitaire SRs with 4 and 6 mm diameters (Table 2; Figure 6). However, despite their unique designs, removal forces with both SRs displayed the same trends when deployed in geometries of varying complexity, requiring higher pulling forces as the arterial model tortuosity increased. These forces increased from 0.24 to 1.20 N between the straight and 3-bend models for the Solitaire Platinum and from 0.37 to 1.55 N for the EmboTrap II. Therefore, within our *in vitro* scenario, the relationship between vessel tortuosity and SR removal force appears to be independent of the SR design. Due to differences in surface properties between our experimental models and cerebral arteries, and differences in anatomical complexity between our 2D geometries and the Circle of Willis, these conclusions may not directly translate clinically. However, we believe our results indicate that vessel tortuosity plays a significant factor in SR retrieval, and should be thoroughly investigated when developing and testing new SR devices.

During their experimental evaluation of multiple commercial SRs, Machi et al. (18) previously reported that larger 6 mm diameter SRs remained in close apposition to the vessel wall during clot retrieval. For this reason, we predicted that the largest diameter SRs evaluated in this study (6 mm) would also have higher removal forces due to greater wall contact resulting in increased frictional forces. By varying SR length and diameter in our work we were able to investigate the influence of these geometric parameters on SR removal force. From this study, no significant differences were observed between devices of the same diameter and varying lengths (i.e., 4 × 15 mm Solitaire 2 and 4 × 20 mm Solitaire Platinum). Comparing SRs of similar lengths and varying diameters (i.e., 4 and 6 mm diameter Solitaire devices), however, revealed significant increases in removal force. Further testing with SRs of greater length variability is needed to fully support our hypothesis that SR diameter is a more influential geometric parameter than SR length with regards to device removal force. In addition, future testing will utilize the most recent iteration of SRs, including the Solitaire X (Medtronic) (47) and Embotrap III (Cerenovus) (48) devices. These SRs have similar strut patterns to their predicates, which lead us to believe that they will exhibit similar removal force magnitudes and trends as those presented in this study.

## 5 Conclusion

SR removal forces were evaluated in experimental models of AIS with commercial SRs of varying diameter, length, and strut design. In addition, simplified arterial models of increasing tortuosity and embolus analogs of varying hematocrit were evaluated as to their effect on SR removal forces. It was found that the hematocrit of the EA occlusion did not significantly influence the measured forces and that, regardless of the SR geometry and design type, the SR removal forces increased with model tortuosity. Further, we hypothesize from the results that SR diameter plays a greater role in the overall removal forces in comparison to SR length, although further testing is needed. These investigations into SR removal forces, which are related to SR-vessel wall apposition and the friction between them, bring us closer to understanding the overall forces involved in MT and potentially increasing rates of FPE. An improved understanding of the parameters necessary for blood clot dislodgement will help to lead to the development of safer and more effective SRs.

## Data Availability

The original contributions presented in the study are included in the article/supplementary material, further inquiries can be directed to the corresponding author.
